# Supplementary material for: Transition metal ion FRET uncovers K+ regulation of a neurotransmitter/sodium symporter
Source: Nat Commun. 2016 Sep 28;7:12755. doi: 10.1038/ncomms12755 (PMC5052704; doi:10.1038/ncomms12755)
Supplement: Supplementary Information — Supplementary Figures 1-5, Supplementary Tables 1 & 2 [file ncomms12755-s1.pdf]

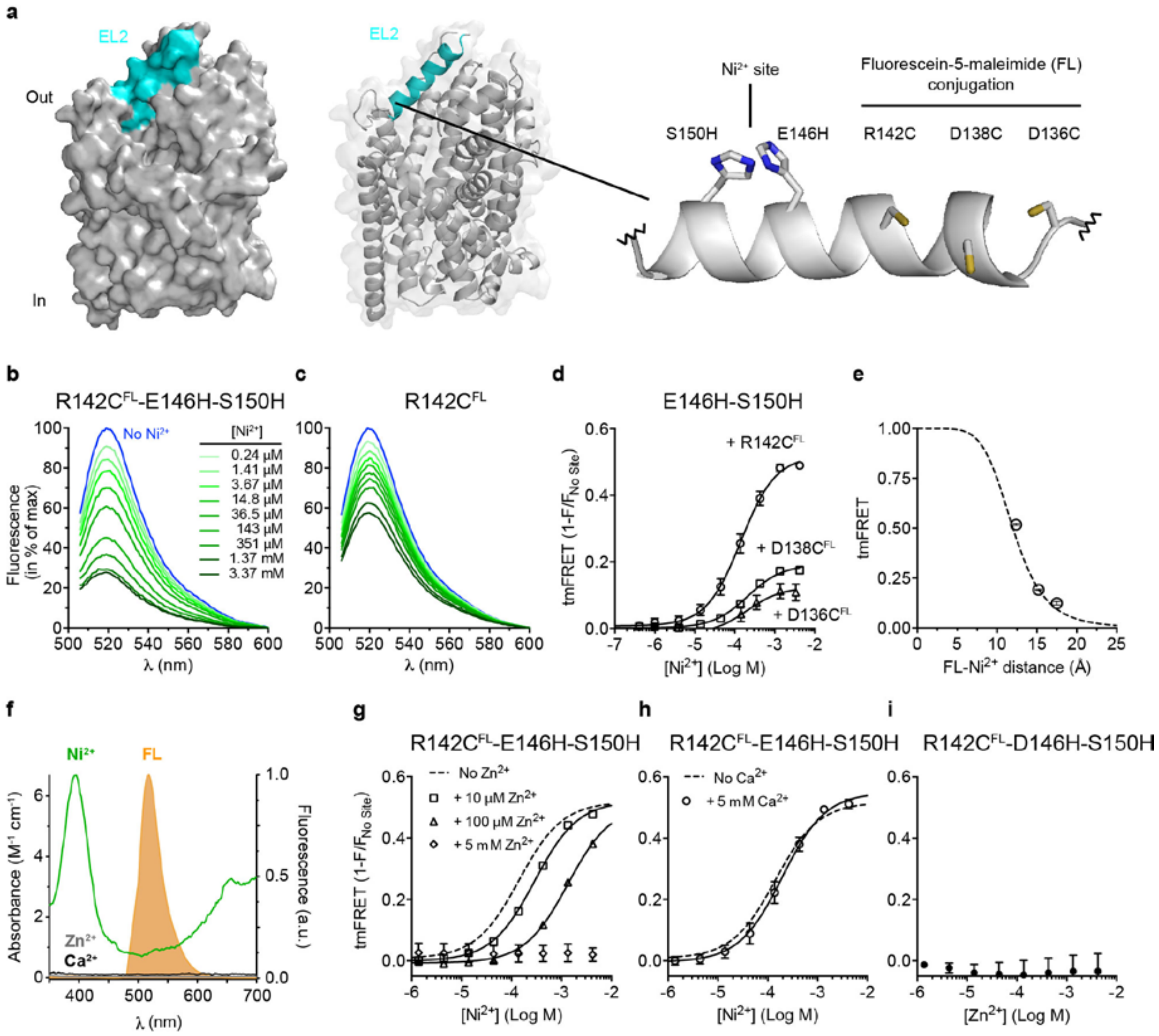

Supplementary figure 1 Application of tmFRET in LeuT. (**a**) To assess the feasibility of using tmFRET for distance-dependent measurements in LeuT, a series of tmFRET-pairs comprised of single cysteine mutants (D136C, D138C, and R142C) labeled with fluorescein (FL), combined with a His-X_3-His motif (E146H-S150H) were generated in the water-exposed α-helical stretch of EL2 (teal, PDB code: 2A65). (**b)** and (**c**) FL-fluorescence was uniformly and dose-dependently quenched by $NiCl_2$. Representative experiment showing that Ni^{2+}-quenching was substantially increased for the mutant harboring a nickel-site (**b**, LeuT R142C^{FL}-E146H-S150H) compared to the control construct (**c**, LeuT R142C^{FL}). (**d**) Specific tmFRET intensity plotted as a function of the Ni^{2+}-concentration in the three LeuT mutants with increasing distance between the cysteine-conjugated FL and the Ni^{2+}-binding His-X_3-His motif (+R142C^{FL} < +D138C^{FL} < +D136C^{FL}, respectively). Maximum tmFRET was inversely related to the donor-acceptor distance for the tmFRET-pair. The EC_{50} of Ni^{2+} for LeuT R142C^{FL}-E146H-S150H was found to be 142 [123;163] μM (mean [s.e.m. interval]). (**e**) The maximum tmFRET values obtained in **d** plotted as a function of donor-acceptor distances estimated from the LeuT crystal structure (2A65). tmFRET data obtained from LeuT follows a Förster distance-relation with R_0 = 12 Å (dotted line), in agreement with previous reports for FL and Ni^{2+}. Data-points are means ± s.e.m. *n* = 3-5. **(f-i)** Specificity of the tmFRET assay. (**f**) The spectral overlap of Ni^{2+}

absorbance and the fluorescence emission of FL. Zn^{2+} and Ca^{2+} are non-colored and, accordingly, show no absorbance. (**g** and **h**) Zn^{2+} but not Ca^{2+} competes with Ni^{2+} for binding to LeuT R142C^{FL}-E146H-S150H. (**g**) Ni^{2+}-titration experiments performed on LeuT R142C^{FL}-E146H-S150H in the presence of Zn^{2+} (10 μM, squares; 100 μM, triangles; 5 mM, diamonds). Zn^{2+} dose-dependently right-shifts the tmFRET curve, suggesting that Zn^{2+} competes with Ni^{2+} for binding to the His-X_3-His site. (**h**) Ni^{2+}-titration experiment performed on LeuT R142C^{FL}-E146H-S150H in the presence of 5 mM Ca^{2+}. The tmFRET curve is unaffected, suggesting that Ca^{2+} does not compete with Ni^{2+}-binding. (**i**) Zn^{2+}-titration experiment performed on LeuT R142C^{FL}-E146H-S150H produced no significant tmFRET. Data points are means ± s.e.m. n = 2-5.

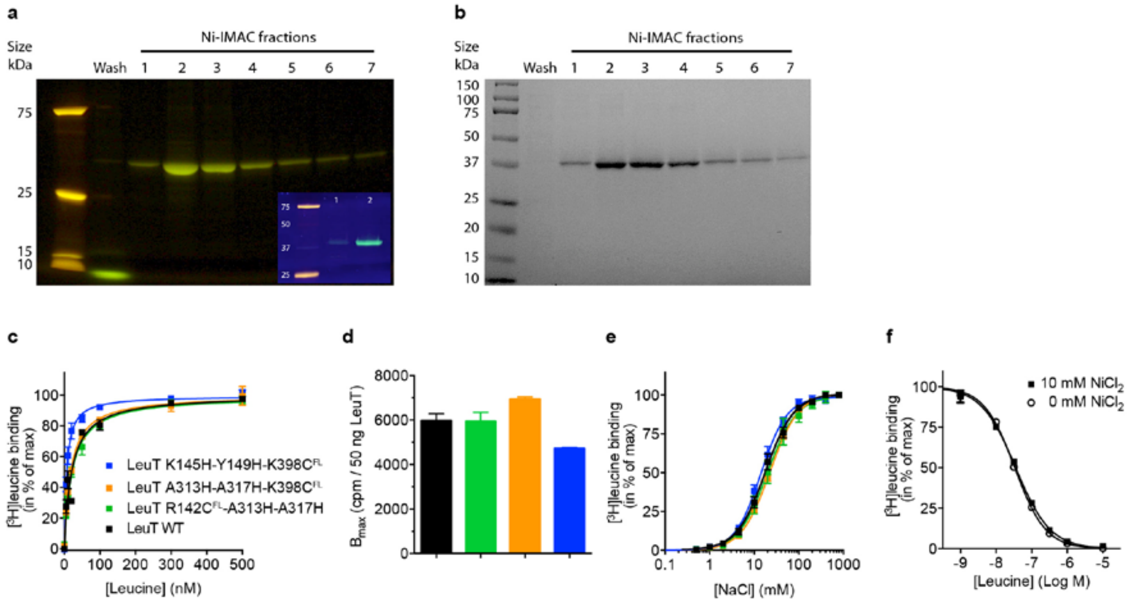

Supplementary figure 2 Purification and functional characterization of tmFRET-mutants. (**a-b**) Representative SDS-PAGE gels with collected fractions (1-7) of Ni^{2+}-IMAC purified LeuT A313H-A317H-K398C^{FL}. (**a**) Fluorescence-scan suggests that conjugation of FL was specific and excess free dye was removed completely by the wash procedure. No fluorescence was observed when eluting purified LeuT WT incubated with fluorescein-5-maleimide (inset: lane 1, LeuT WT; Lane 2, LeuT K398C). (**b**) Coomassie staining showing sample purity of LeuT at ~37 kDa. (**c-e**) The generated tmFRET-pairs retained wild type-like substrate- and sodium-binding affinities. (**c**) Saturation binding of [^{3}H]leucine measured by scintillation proximity assay (SPA) in the presence of 200 mM NaCl and 5 mM $NiCl_2$ for LeuT K145H-Y149H-K398C^{FL} (blue), LeuT A313H-A317H-K398C^{FL} (orange), LeuT R142C^{FL}-A313H-A317H LeuT (green), and LeuT WT (black). (**d**) Total binding sites (B_{max}) measured with 500 nM [^{3}H]leucine in 200 mM NaCl and 5 mM $NiCl_2$, labels are the same as in **c**. (**e**) Na^+-stimulated binding of 100 nM [^{3}H]leucine in the presence of 5 mM $NiCl_2$, substituting KCl for NaCl. Color labels are the same as in **c**, and WT LeuT was assayed in the absence of $NiCl_2$. (**f**) Homologous competition binding of 10 nM [^{3}H]leucine and leucine to LeuT WT, in 0 mM $NiCl_2$ (open symbols) or 10 mM $NiCl_2$ (filled symbols). Data-points are means ± s.e.m., *n* = 3-4

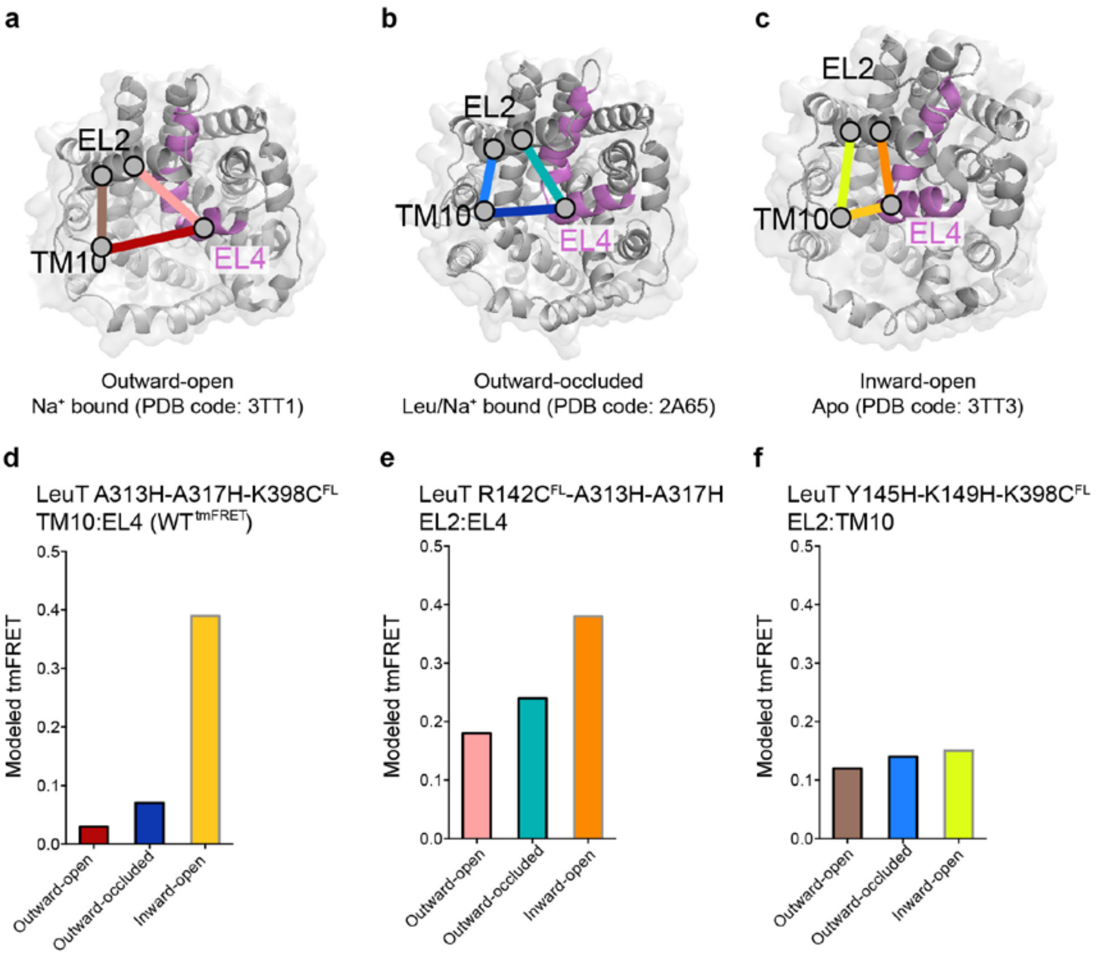

Supplementary figure 3 Evaluating structural implications of tmFRET measurements. Crystal structures viewed from the top of LeuT in (**a**) outward-open (Na^+-bound, PDB 3TT1), (**b**) outward-occluded (Na^+ and leucine bound, PDB 2A65), and (**c**) inward-open (apo, PDB 3TT3) states. Positions of the generated tmFRET pairs are shown in the cartoon representations of the crystal structures (grey circles). The solid lines indicate the donor-acceptor distances between C_β-atoms of inserted cysteines and His-X_3-His motifs. (**d**) Bar-graph shows the TM10-EL4 (LeuT A313H-A317H-K398C^{FL}) tmFRET values which would be expected if the actual distances were as measured from the crystal structures above (**a-c**). The colors of the bars correspond to the colors of the distances (solid lines) between the tmFRET probes (grey circles) in **a**, **b** and **c**. (**e**) Bar-graph with predicted EL2-EL4 tmFRET intensities based on measured distances (solid lines) in LeuT structures as for **d**. (**f**) Bar-graph with predicted TM10-EL2 tmFRET intensities based on measured distances (solid lines) in LeuT structures as for **d**.

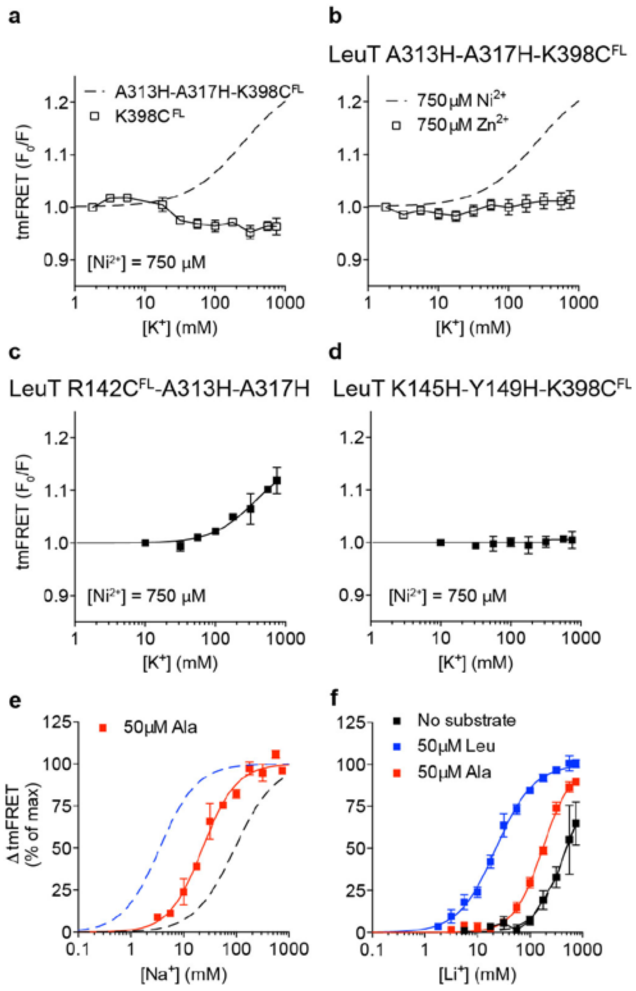

Supplementary figure 4 Specificity of ions and substrates on the tmFRET response. (**a**) Effect of KCl on the tmFRET background mutant LeuT $K398C^{FL}$ (with 750 μM Ni^{2+}), suggesting that KCl has no apparent effect on FL emission in the absence of the His-X_3-His motif. LeuT A313H-A317H-$K398C^{FL}$ from **Fig. 3a** is shown as dashed line for reference. **(b)** Increasing KCl in LeuT A313H-A317H-$K398C^{FL}$ performed with 750 μM Zn^{2+} does not change tmFRET, suggesting that Ni^{2+} is required for a positive tmFRET response by K^+. Dashed line shown for reference, is same as in **a**. Data are means ± s.e.m. *n* = 3-4. (**c**) K^+ titration for LeuT $R142C^{FL}$-A313H-A317H (EL2:EL4) performed as described in **b** and fitted to the Hill equation yielding an EC_{50} = 294 [109;794] mM. (**d**) K^+ titration for LeuT K145H-Y149H-$K398C^{FL}$ performed as described in **b**. Data points are means ± s.d. *n* = 2-4. (**e**) Monitoring tmFRET between TM10 and EL4 (LeuT A313H-A317H-$K398C^{FL}$) in response to Na^+ in the presence of 50 μM alanine (red squares), in buffer with 600 mM KCl and 750 μM Ni^{2+}. The effect of Na^+ in the absence of substrate (black dashed line) and 50 μM leucine (blue dashed line) are shown for reference, both from **Fig. 3d**. (**f**) tmFRET between TM10 and EL4 (LeuT A313H-A317H-$K398C^{FL}$) in response to Li^+ in the presence of 50 μM alanine (red squares), 50 μM leucine (blue squares), or in the absence of substrate (black squares). Experiments in **e** and **f** were carried out in buffer with 600 mM KCl and 750 μM Ni^{2+}, and data points are means ± s.e.m. of 4-5 independent experiments in Li^+ and two independent experiments in Na^+. Binding constants are shown in Table 1.

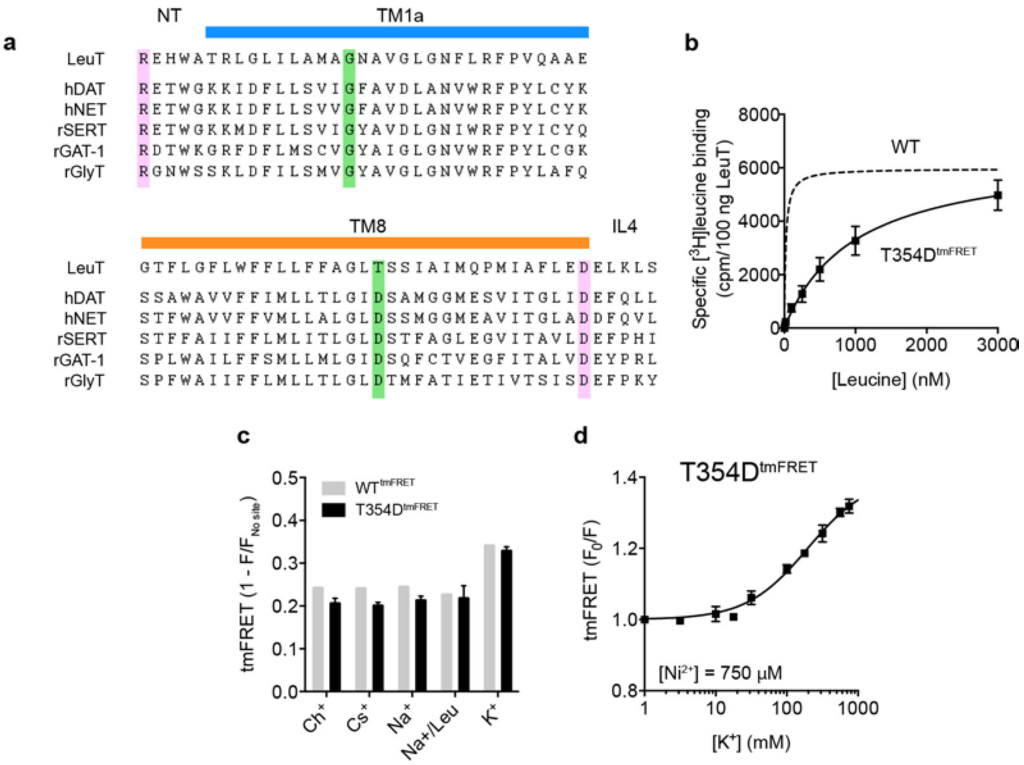

Supplementary figure 5 The effect of K^+ on tmFRET response is preserved in T354D. Converting the Na2-site in LeuT to corresponding residues in mammalian NSS members (T354D) decreases leucine affinity but recapitulates WT^{tmFRET} results. (**a**) Sequence alignment of the N-teminal (NT), TM1, and TM8 between LeuT and mammalian NSS members. Position of the intracellular salt-bridge (red; R5, D369) and the Na2-site ligands (green; G20, T354) are indicated. (**b**) SPA saturation binding of [^{3}H]leucine to $T354D^{tmFRET}$ (LeuT A313H-A317H-T354D-$K398C^{FL}$) in 200 mM Na^+ (EC_{50} = 995 ± 70 nM) (**c**) tmFRET in $T354D^{tmFRET}$ (black bars) relative to WT^{tmFRET} (grey bars) in 200 mM of the indicated ions shows no difference between constructs. (**d**) tmFRET change in response to K^+ for $T354D^{tmFRET}$ in buffer containing 750 µM $NiCl_2$. Data fitted to the Hill equation (EC_{50} = 198 [131;299] mM; n_{Hill} = 1.01 ± 0.10) showed similar affinity and slope as observed for WT^{tmFRET} (see **Fig 3a**). Data points are means ± s.e.m. *n* = 3-6.

Supplementary Table 1 Radiotracer binding constants for LeuT WT and tmFRET variants

LeuT variant	K_D (nM)	B_{max} (% of WT)	EC_{50} Na^+ (mM)	n_{Hill}
WT	20.1 ± 4.2	100	20.1 [17.5;23.2]	1.36 ± 0.09
R142C^{FL}-A313H-A317H	21.3 ± 1.8	99.4 ± 12	19.6 [16.2;23.8]	1.33 ± 0.16
K145H-Y149H-K398C^{FL}	6.63 ± 0.7	79.0 ± 4.2	14.1 [12.7;15.7]	1.49 ± 0.11
A313H-A317H-K398C^{FL} (= WT^{tmFRET})	17.8 ± 1.3	116 ± 6.2	22.5 [21.3;23.7]	1.35 ± 0.04
T254V^{tmFRET}	95,000 ± 2,700§	~ 80§	-	-
T354V^{tmFRET}	5,450 ± 400§	104 ± 7.6§	-	-
´T354D^{tmFRET}	995 ± 70	111 ± 6.6	-	-

K_D and B_{max} determined by saturation [^{3}H]leucine binding in the presence of 200 mM Na^+. EC_{50} for Na^+ stimulated binding of 1 µM [^{3}H]leucine binding determined by substitution with K^+ or Ch^+. Data shown as means ± s.e.m. or [s.e.m. interval], *n* = 3-4. § Assayed in 800 mM NaCl

Supplementary Table 2 tmFRET efficiencies for tmFRET variants

tmFRET variant	Na^+	Na^+/Leu	K^+	Ch^+	Cs^+
WT^{tmFRET} (= EL4-TM10)	0.244 ± 0.008	0.227 ± 0.005	0.341 ± 0.006	0.242 ±0.010	0.241 0.009
R30A^{tmFRET}	0.109 ± 0.004	0.148 ± 0.004	0.091 ± 0.003	n.d.	0.079 ± 0.007
T254V^{tmFRET§}	0.303 ± 0.005	0.294 ± 0.005	0.343 ± 0.004	0.272 ± 0.018	0.270 ± 0.010
T354V^{tmFRET§}	0.444 ± 0.004	0.325 ± 0.012	0.444 ± 0.004	0.430 ± 0.020	0.457 ± 0.016
T354D^{tmFRET}	0.213 ± 0.010	0.261 ± 0.022	0.328 ± 0.010	0.206 ± 0.012	0.201 ± 0.008
EL2-EL4	0.172 ± 0.008	0.229 ± 0.007	0.264 ± 0.005	0.179 ± 0.02	0.177 ± 0.010
EL2-TM10	0.137 ± 0.008	0.170 ±0.004	0.151 ± 0.007	0.143 ± 0.009	0.127 ± 0.007

Experimentally measured tmFRET efficiencies for the investigated tmFRET variants: WT^{tmFRET} (EL4-TM10, or A313H-A317H-K398C^{FL}) and mutations in this background (R30A, T254V, T354V and T354D), EL2-EL4 (R142C^{FL}-A313H-A317H) and EL2-TM10 (K145H-Y149H-398C^{FL}). Values are maximum tmFRET values (5 mM Ni^{2+}). §Buffers containing 800 mM of the indicated cation. n.d.; not determined.
